# Football to eyeball: thinking out of the box to create an ophthalmic talent pool in difficult geographies

**Published:** 2018-07-31

**Authors:** Mritunjay Tiwary

**Affiliations:** 1 Founder & Head of Projects: Akhand Jyoti Eye Hospital, India.

**To create and retain locally available pool of ophthalmic talent, thinking out of the box has helped Akhand Jyoti Eye Hospital to recruit, train and retain local talent successfully.**

**Figure:**
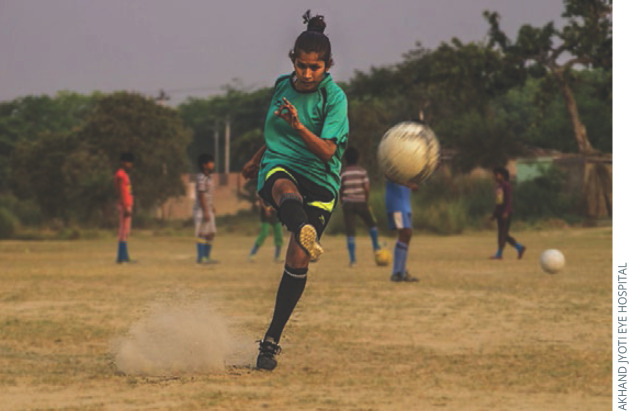
Football is used as an icebreaker to negotiate opportunities for young girls. INDIA

It is well established that a pool of optometrists and similar allied ophthalmic personnel are the backbone of any successful eye care programme. It is also true that recruiting and training them is a significant challenge; high turnover of such human resources makes the problem even more significant. Similar scenarios under challenging geographies, especially areas of high poverty and poor infrastructure, makes it all the more complicated.

To create a locally available pool of ophthalmic talent, we need to think beyond traditional solutions. Thinking out-of-the-box has helped us at Akhand Jyoti Eye Hospital to recruit, train and retain local talent successfully.

The hospital was started in early 2006 in a remote village in the eastern Indian state of Bihar. It grew from a ten-bed hospital to 400 beds and from 4000 annual surgeries to 65,000 surgeries in just seven years. All the while, it never had to struggle for ophthalmic personnel, especially optometrists and ophthalmic assistants.

The “football to eyeball” programme was started in 2010 to develop a local talent pool of ophthalmic personnel. Under this programme, girls from the local villages are encouraged to play football and train to qualify as professional optometrists thereby empowering them to cure blindness and make a broader societal impact in a very patriarchal society.

This unique programme uses football as an icebreaker to negotiate opportunities for young girls. Girls between the ages of 12-16 are nurtured by the hospital to aspire to become professional footballers or optometrists or both. This initiative is instrumental in targeting gender-based inequalities, exploitation and child marriage - all of which afflict girls in Bihar – and to provide equal opportunities to them. We work as a hub-and-spoke model wherein football is a crucial instrument of change. Our motto for the programme is “teach football to the girls and draw them out of their homes.” The eye hospital works as a hub for this programme. The spokes are the villages where we had conducted outreach camps.

Football is introduced as a sport to these girls under the supervision of a physical instructor in the local government schools of the villages. Once a girl develops an interest in the sport, we offer them to join full-time and reside at the hostel facilities within the hospital centre. The entire cost of education, training, and living is undertaken by Akhand Jyoti with an objective to motivate these girls to become ophthalmic personnel and role models for future in their local communities.

The girls can simultaneously opt for a four-year bachelors course in optometry after completing their standard XII (A levels). As a qualified optometrist, she can choose to practice at the hospital or start her own optical clinic. This qualified optometrist can easily earn at least five times more than the per capita earning of the rural families in India, creating significant opportunities for livelihood and improving gender parity in the society. Over 90% of these girls opt to work full-time at the hospital centres thereby creating a vast pool of talent from which we can choose. The hospital's current and future human resources gaps mandated stability in support staff, especially ophthalmic personnel. The football to eyeball programme helped us achieve this and at the same time helped us address a significant social issue in the local community – gender inequality.

In summary, these girls are enrolled and provided secondary education, including English and computing skills, and then trained on the optometry and ophthalmic assistant course, and finally offered employment in the hospital. The optometry course combines theory and practical sessions conducted by our in-house ophthalmologists and senior optometrists. The completion of the course ensures that the girl is qualified to practice as an optometrist and in turn they can assist Akhand Jyoti Eye Hospital achieve its vision of eliminating blindness in low-income states of India.

**Figure 1:**
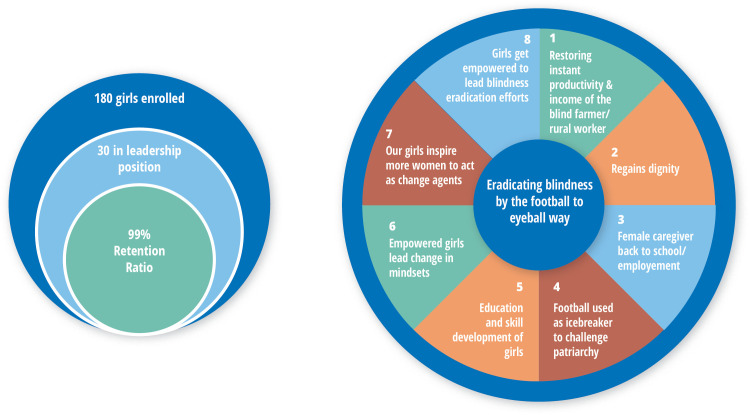
The success of the programme and the link with blindness.

## Key learnings from this talent creating exercise are

- The personal commitment of the leadership towards talent creation is crucial.
- Locally available talent is more straightforward to retain; much more so if their training is carried out at the institution where they are employed.
- Eye care programmes have the potential to make a more significant social impact on local communities.
- Solutions to human resource issues are hidden in the community themselves; it is just a matter of understanding how to transform and use the available raw talent.

**Figure:**
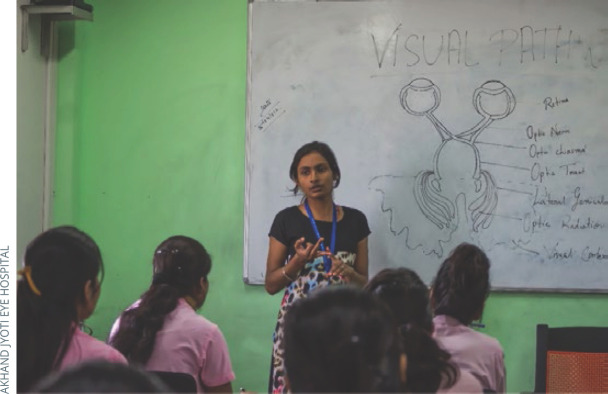
Optometry class in progress. INDIA

## A similar model can be replicated by applying the following steps

- Primary objective (critical) – identify an inequity or unjust situation which exists in the community (we identified the girl child – they face strong gender, social and economic inequality in Bihar).
- Secondary objective – to create a locally available pool of skilled manpower which the organisation can continuously use
- Identify main skills desired to be imparted (optometry in our case)
- Identify the bridging abilities (for us it was English and computing skills)
- Identify how and where the career opportunities at the local level would come from (for us direct employment at the hospital).
- Formalise the enrollment process (we documented the agreements, career plan, sponsorship mechanism, and exit policies)
- Start with direct contact with parents and counsel them along with the candidates (it took us nine months to convince the first girl to enrol; now we have waiting lists of 700 names).
- Identify and create an enabling environment (we had greater success when girls started living 24×7 with us rather than the earlier situation when they were with us for three days in a week)
- Identify the talent with preference to those living in greater inequity (our primary selection criterion is the economic condition of the family).
- Groom and nurture the talent (our priority for the first three months was to improve confidence levels and change mindsets)
- Create a long-term plan to retain the talent (we devised and communicated, in advance, a three year career plan after the girls completed their course)

